# Validation of a Textile Material’s Electrostatic Characterization Device for Different Parameters and Their Effect on the Electrostatic Charge Generation

**DOI:** 10.3390/ma15165716

**Published:** 2022-08-19

**Authors:** Hasan Riaz Tahir, Benny Malengier, Didier Van Daele, Lieva Van Langenhove

**Affiliations:** Centre for Textile Science and Engineering, Department of Materials, Textiles and Chemical Engineering, Ghent University, Zwijnaarde, 9052 Ghent, Belgium

**Keywords:** textile material’s electrostatic charges, characterization of motion, electrostatic waveform, peak voltage, peak-to-peak voltage

## Abstract

This research aims to validate an electrostatics characterization device to better understand the process of static charge generation in textile materials and to see how different factors affect it. This electrostatic device offers a variety of settings for controlling sample electrostatic activation and has a sample size range of up to one square meter. It can move in both horizontal and vertical directions in a controlled manner, providing a variety of possibilities for testing the effect of various movement features on electrostatic charge formation. Not only the textile polymer but also the motion characterizations influence the generation of electrostatic charges in textiles. The influence of frequency, pressure, dwell time between moves, test duration, effect of different sample sizes, and amplitude of movement on electrostatic charge generation was studied in greater detail. Two different parameters of the electrostatic waveform (peak voltage and peak-to-peak voltage) were investigated. The generation of electrostatic charges is proportional to the peak voltage and peak-to-peak voltage of the electrostatic waveform. Overall electrostatic charge generation increases with increasing frequency, stepping height, applied pressure at the same frequency, and sample size, but decreases with increasing dwell time between moves at the same frequency. The charge also increases with test duration until a saturation point is reached.

## 1. Introduction

The measurement of electrostatic charge generation in textile materials can be done for two reasons. The first is to determine its generation to create better textile cloths [1] and floorcoverings with less static buildup [2], while the second is to optimize it to capture the Nano energy and utilize it to charge low-power sensors and electronic devices [3,4]. Concerning the last reason, it is vital to characterize them in order to maximize the charge generation for energy harvesting [5,6,7] and see the various parameters that control it. A device for determining the electrostatic behavior of textiles and floorcovering has been validated [8].

Electrical charges are the fundamental component of all static electricity. These come in both positive and negative. Atoms, which make up all materials, contain these electrical charges. Atoms have a positively charged nucleus and negative electrons around the nucleus. When these charges are present in about equal amounts, which they typically are, their electrical effects are neutralized. A substance may occasionally have a slight excess of positive or negative charges; in this instance, we refer to the material as being charged, and static electrical phenomena may be observed [9,10,11]. Triboelectrification, a method of contact and frictional charging, can be used to separate these charges, which are present in all materials [12,13,14]. A small number of electrons transfer from one substance to the other when any two materials come into contact. The imbalance of charge is carried by each material if the elements are separated afterward. The materials are charged, with one having an excess positive charge and the other having an equal number of negative charges. Only eight atoms per million on the surface are needed to achieve the maximum charge density of a surface, which is constrained by the air breakdown field strength, in order to produce powerful static electricity effects [15,16,17,18].

The static electricity that builds up on textiles during use is mainly caused by friction and tapping [19,20]. People become uneasy when their garments cling to their bodies or feel like they are crawling due to static electricity. When putting on or taking off garments, the most common and irritating electrostatic discharge problem arises, which can cause uncomfortable and restless symptoms [21]. Electrostatic charging also has physiological impacts on the human body. Electrostatic charge is a truth that we can observe in our daily lives at any time. For example, static electricity can charge the human body while walking on a floorcovering, rising from an armchair, or removing clothing. In addition, an electrostatic discharge occurs when a person charged with static energy meets a metal object, and there may be a spark at the point of contact [22,23,24]. The material type, moisture content of the test material, relative humidity and temperature of the environment, and the number of repetitions of the rubbing movement are all essential elements that affect electrostatic characteristics. Aside from this, extrinsic influences might significantly impact textiles’ electrostatic propensity. Various studies on the static electrification of textile goods and the factors affecting static electrification have been conducted [23,25,26].

Static electric charges have been a major problem especially for products developed from manmade fiber. Extensive research on the measurement devices and procedures has been done to understand the mechanism of generation of electrostatic charges and to understand the complicated and often not reproducible results [21,27,28,29]. The amount of build-up of charge depends not only upon different material parameters [14,30,31,32] but also on the characteristics of motion [26,28,33,34]. The goal of this research is to have a better understanding of the generation of electrostatic charges and to see the effect of different parameters on its generation. Significant research has been performed, and several thorough evaluations of the electrostatic charging mechanism on textile materials have been published [14,35,36,37]. 

Contact Electrification (CE) between two dissimilar materials results in the production of triboelectric charges on surfaces. The contribution of the electrostatic charges caused by contact electrification in the triboelectric energy harvesting is given in the Maxwell’s equations [18,38]. Wang and colleagues first developed the TENG to generate electricity out of low-frequency sources. TENG uses the coupling effect of electrostatic induction (ESI) and contact electrification (CE), and it benefits from simple production, a wide range of material options, and a variety of applications [39,40]. Whether or not nanomaterials are used, TENG is a field that effectively converts mechanical energy into electric power. Recently, triboelectric nanogenerators (TENGs) have emerged as a potent technology for self-powered sensor networks and energy harvesting. Their ability to work with a variety of materials, especially when creating affordable and simple-to-use devices, is one of their main advantages [41,42]. The textile triboelectric nano generator (TENG) is a type of smart textile technology that combines conventional flexible and wearable textile materials. The TENG embraces the capabilities of autonomous energy harvesting and active self-powered sensing but still preserves the original wearability and desired comfortability. People may easily acquire and efficiently use electric energy with the aid of a self-sufficient wearable intelligent system, which will support the advancement of human-oriented on-body electronics and artificial intelligence in the future [43,44,45]. Here, some important key parameters were investigated that effect the electrostatic charges generation during the contact and separation motion. The device could not only control the track of horizontal motion but also different attributes of vertical motion. 

However, the current study is also particularly important since it explains the different characteristics of motions and their effect on the generation static charges, which is especially useful in optimization of electrostatic characterization parameters for electrostatic testing of floorcoverings and energy harvesting from electrostatic charges. This research is especially to validate a device that can walk on floorcovering to check its electrostatic behavior, but also the vertical movement could be precisely controlled. Additionally, this device is unique for the use of a sample size up to 1 m^2^ with the movement of the second sample with specific controlled movement above it. This movement could be controlled with not only contact time but also dwell time (time between moves) and XY (horizontal) direction. The combination of all the possible parameters (frequency, pressure, testing time, stepping height, dwell time, and sample sizes) is controlled within one setup.

## 2. Materials and Methods

**Figure 1:**
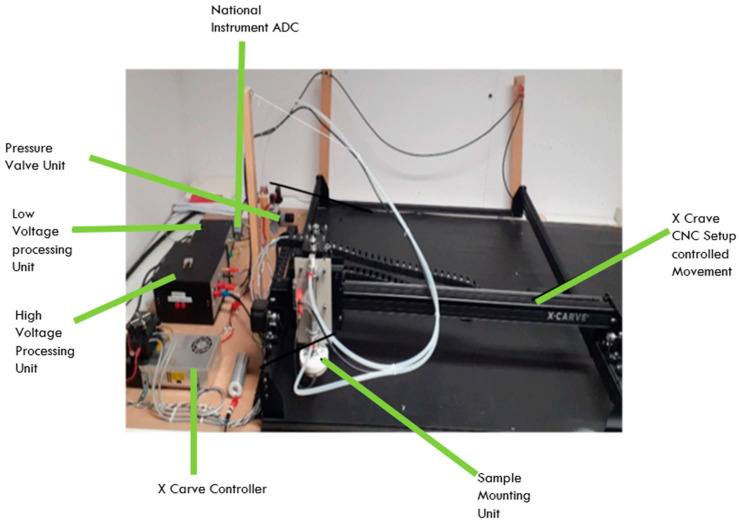
Different components of electrostatic characterization device.

**Figure 2:**
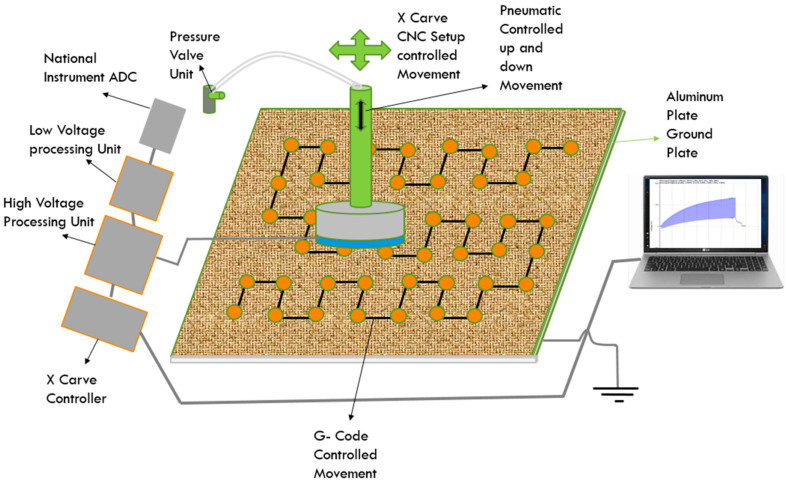
Schematic diagram of electrostatic characterization device.

The material’s electrostatic characterization device consists of different parts as seen in Figure 1 and illustrated in the schematic diagram in Figure 2. In the middle is the X-Carve, where the movement on the samples takes place. On the shelf is an aluminum plate, which is connected to the ground. This plate, therefore, serves as a reference for the measurements. A plastic mat is placed on the metal plate, which ensures insulation of the carpet in relation to the reference plane. These two layers remain permanently in the system. The piece of textile to be tested can then be placed on the plastic mat. On the left side of the X-Carve are all the electronics, controls, and pneumatics. At the bottom of the figure are the power supply and control of the X-Carve. Above that are both electronic boxes and the analog-to-digital converter (ADC) used to record the waveform. The pneumatic circuit is located behind the electronics. A standard compressed air connector is provided to connect the compressed air to the pressure regulator. The regulated pressure then flows to the valve, which is actuated by the control of the X-Carve. The compressed air lines are directed upwards to prevent the lines from sinking down. The pneumatic cylinder, which acts as an actuator for up and down motion, was fixed to the Z-axis of the X-Carve via an adapter plate. In this way, the step height can be set from the GUI (Graphical User Interface).

**Figure 3:**
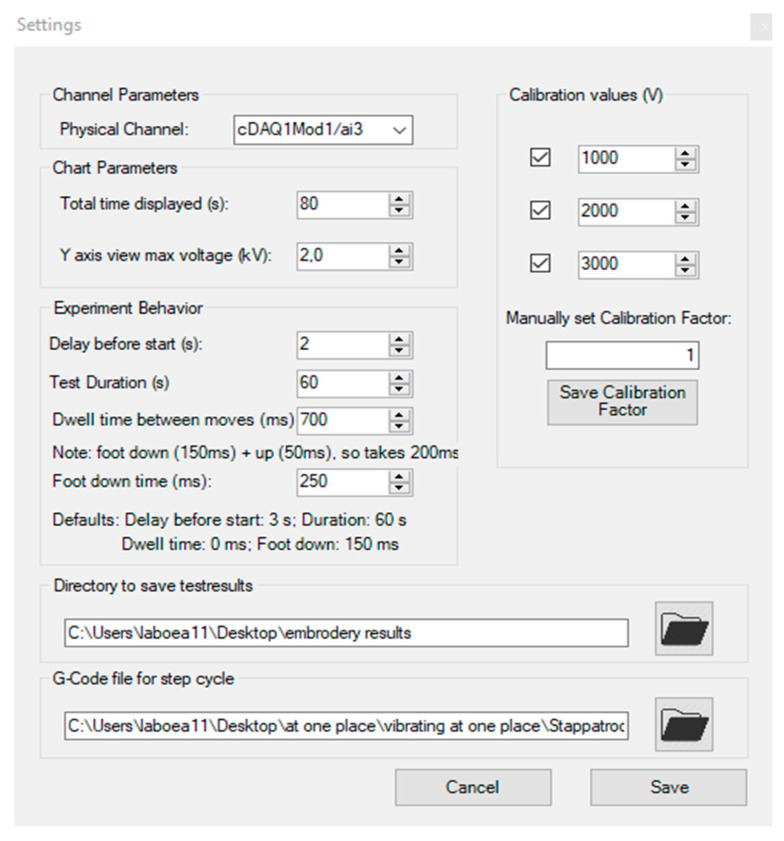
The GUI (Graphical User Interface) shows the control of total test time, 1 hertz frequency with help of Dwell Time (700 ms) + Foot Up Time (50 ms) + Foot Down Time (250 ms).

The GUI (Graphical User Interface) is shown in Figure 3 and is used to precisely control the total test time, dwell time, foot up, and foot down time. The effect of electrostatic charge generation has been studied at three different frequencies. In order to obtain the 1, 1.5, and 2-hertz frequency, the following settings have been used for the different time parameters.

1. 1 Hz Frequency: Foot Down Time (250 ms), Dwell Time (700 ms), Uptime (50 ms)
2. 1.5 Hz Frequency: Foot Down Time (250 ms), Dwell Time (450 ms), Uptime (50 ms)
3. 2 Hz Frequency: Foot Down Time (250 ms), Dwell Time (200 ms), Uptime (50 ms)

Three different pressure-applied settings of 0.5, 1, and 1.5 bar were selected with the help of a pressure valve unit to study the effect of pressure applied on the electrostatic charge generation. The effect of testing time was studied at six different test duration times: 10, 20, 30, 40, 50, and 60 s selected with the option given in GUI (test duration). Three different distances (10 mm, 30 mm, and 50 mm) between the up and down movement were selected at the same frequency, applied pressure, and total test duration. For dwell time between moves, three different times—100 ms, 200 ms, and 300 ms—were selected at 2 Hz frequency. Three different sample sizes were selected to study the effect of electrostatic charge generation for the same material and the same setting of applied pressure, frequency, stepping height, and test duration. The maximum peak voltage and peak-to-peak voltage were calculated and given in tables to see the effect of different parameters on electrostatic charge generation. The floorcovering pile fiber composition was 100% PA6, with bitumen backing, loop pile, nominal mass of 4128.4 g/m^2^, and total thickness of 6.9 mm.

The tests were carried out using the new device and with a one-square-meter sample size, with BAM as the sole material for the testing related to effect of sample size and conductive copper fabric (Shieldex^®^, Bremen, Germany) for the rest of the parameters (frequency, dwell time, pressure, testing time, stepping height). The sample was cut to three different sizes to examine the influence of sample size. Three repetitions were performed for each parameter (frequency, dwell time, pressure, testing time, stepping height, and sample size). The average highest peak voltage was calculated for each repeat by averaging five peaks and valleys as described in standard ISO 6356. The difference between the highest peaks and highest valleys on an electrostatic voltage graph was used to compute the peak-to-peak voltage.

## 3. Results and Discussion

### 3.1. Effect of Frequency

**Figure 4:**
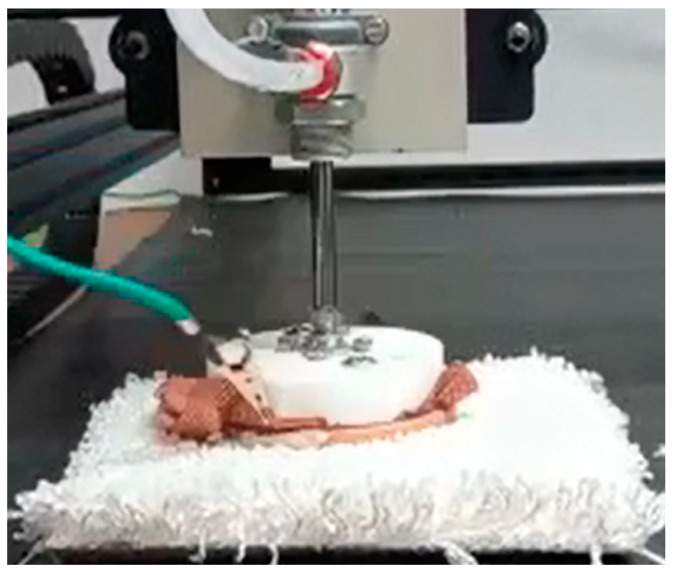
Compressible floorcovering sample to see the effect of variable pressure.

**Figure 5:**
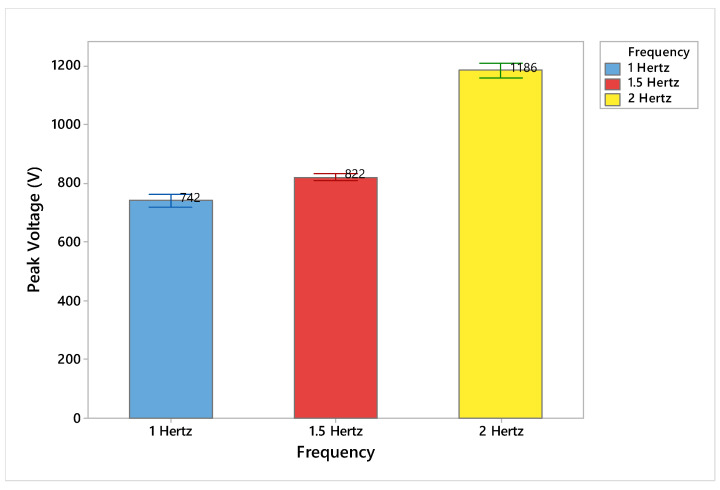
Effect of different frequency (Hertz) on the electrostatic charge generation.

**Figure 6:**
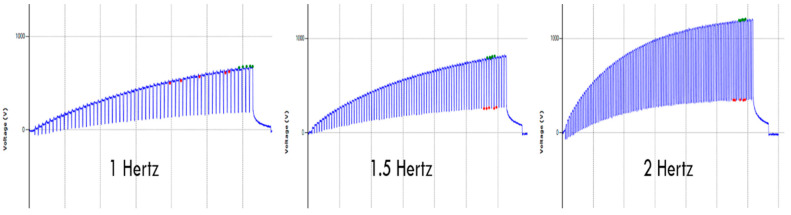
Effect of different frequency on the electrostatic waveform, peak voltage, and amplitude (peak-to-peak voltage).

The effect of three different frequencies—1 hertz, 1.5 hertz, and 2 hertz—on the electrostatic charge generation are summarized in Table 1. In Figure 4, the output voltage (peak voltage, peak-to-peak voltage) was increased with increase in frequency from 1 Hz to 2 Hz. The increase in voltage is due to more contact and separation motions that generate more electrostatic charges, and hence more output voltage. There is an increase in the electrostatic charge generation if we increase the frequency of tapping between the samples by taking the contact time constant indicated by the increase in peak voltage and peak-to peak-voltage, as shown in Figure 5 and Figure 6. In addition, with the increase in frequency, there is an increase in the speed of movement and contact time that generates more electrostatic charges at the same pressure, stepping height, and testing time. The coefficient of variation (CV %) is the ratio of the standard deviation to the mean expressed as percentage. 

### 3.2. Effect of Different Pressure

**Figure 7:**
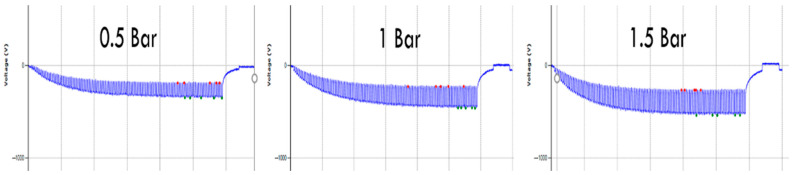
Effect of different pressure on the electrostatic waveform.

The effect of applied pressure is more significant for compressible textiles or floorcoverings, as shown in Figure 7, and hence have a different area of contact at different pressure. A floorcovering sample with bulky piling has been selected to see the charge generation at different pressure as shown in Figure 4. Three different pressures (0.5, 1, and 2 bar) have been applied to see the effect of changing pressure on electrostatic charge generation (see Table 2). There is an increase in peak voltage and peak-to-peak voltage with the successive increase in applied pressure. The output voltage increases more when the pressure applied is changed from 0.5 to 1 bar as compared to 1 bar to 1.5 bar. The increase in static charges with increasing pressure is due to the increased compressing of the textile structure, which causes more inter-fiber friction. Over one bar, the change in static charges is not so large anymore, so over a threshold pressure, the influence of pressure will not be large anymore.

### 3.3. Effect of Test Duration Time

**Figure 8:**
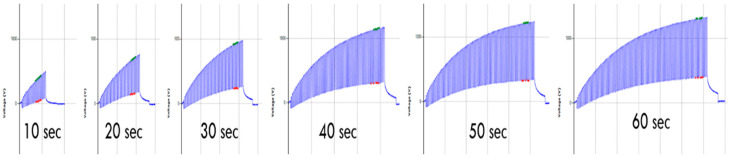
Time duration effect on electrostatic waveform to get a saturation point.

**Figure 9:**
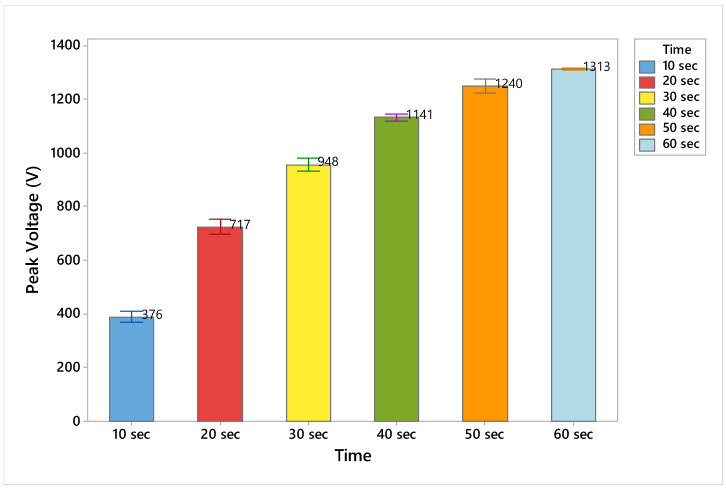
Effect of testing duration on the electrostatic charge generation.

The test duration is essential to see a saturation point in the electrostatic charge generation waveform. Table 3 summarized the effect of different test durations (10 s, 20 s, 30 s, 40 s, 50 s, 60 s) on the electrostatic charge generation. There is a successive increase in peak voltage and peak-to-peak voltage with the increase in total test time, as shown in Figure 8 and Figure 9. At 60 s, the saturation is almost reached, and a balance must be found to allow sufficiently fast testing. While the time increased from 40 s to 50 s, the increase in output voltage was 10%, and it was just 5% from 50 s to 60 s.

### 3.4. Effect of Stepping Height

**Figure 10:**
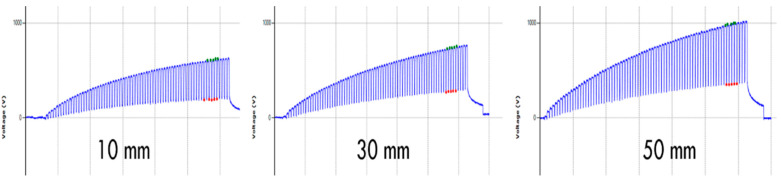
Effect of different stepping height on the electrostatic waveform.

The effect of different stepping heights of 10, 30, and 50 mm on the electrostatic charge generation is summarized in Table 4. There is an increase in peak voltage and peak-to-peak voltage with the increase in stepping height from 10 mm to 50 mm, as shown in Figure 10. As the frequency is the same for each stepping height, there is an increase in speed of movement, which corresponds to the higher generation of electrostatic charges. Compared to human testing, this might explain some of the differences between human testing and device testing, as humans will take steps that typically lift the foot more than 50 mm. The increase with height is also significant with a rise of 32% when going from 30 to 50 mm, so large differences can arise if humans step on the same carpet.

### 3.5. Effect of Different Dwell Time between Moves at Fixed Frequency

**Figure 11:**
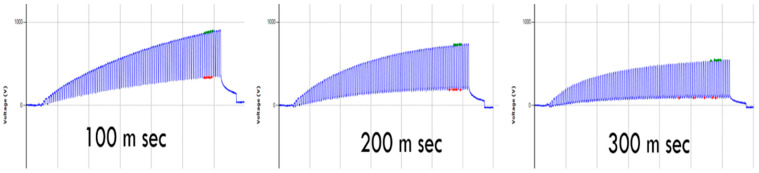
Effect of dwell time on the electrostatic waveform.

The dwell time is the time between the up and down maximum point. Table 5 shows the effect of dwell time on the electrostatic charge generation at a stepping frequency of 2 Hz. It was found that there was a decrease in electrostatic charge generation with the increase in dwell time. As the movement frequency is the same, the increase in dwell time corresponds to a decrease in movement time (higher speed), generating fewer electrostatic charges at higher dwell time, as shown in Figure 11.

### 3.6. Effect of Sample Size

**Figure 12:**
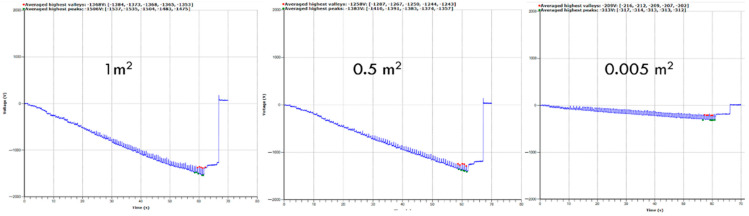
Effect of different sample size on the electrostatic waveform.

The study of electrostatic charge generation was tested for the different sample sizes to see the effect of exposed surface area on the generation of electrostatic charges. For this, three samples size of the same materials were selected, as summarized in Table 6. The increase in sample size increases peak voltage and peak-to-peak voltage. The reason is bigger surface area means more exposure to material that has not yet been stepped on. So, the material where stepping occurs is not charged yet and can further charge the foot. Figure 12 shows the increase in electrostatic charge generation with the increase in sample size. It is clear that over a specific size, there is sufficient material to step on, leading to a cut off of how much charge is possible as the sample size increases.

## 4. Conclusions

Six parameters (frequency, pressure, testing time, stepping height, dwell time, and different sample sizes) could be controlled with the automatic electrostatic characterization device. The effect of different parameters on electrostatic charge generation was studied with the help of peak voltage and peak-to-peak voltage. Peak voltage increases with the increase in frequency, stepping height, time duration of the test until a saturation point, applied pressure at the same frequency, and sample size, but decreases with an increase in dwell time at the same frequency. Additionally, the peak-to-peak voltage increases with the increase in frequency, stepping height, time duration of the test until a saturation point, and applied pressure at the same frequency, but decreases with an increase in dwell time at the same frequency. Therefore, various motion-related parameters controlled through this device were validated by the peak and peak-to-peak voltage to see their effect on the overall output voltage. 

Higher frequency causes more electrostatic charges to be generated due to faster movement. The higher frequency is achieved by reducing the dwell time, so a shorter dwell time leads to more electrostatic charge. This corresponds to what is seen when the frequency is kept fixed, but the dwell time increases. This increase in dwell time leads to a decrease in electrostatic charges. We can conclude that contact and release generate static charges, which slowly disappear again as the materials remain in contact. So, the dwell time will have a high effect on the result and should be well-defined when doing automated static testing. As the stepping height increases, more electrostatic charges are generated due to increased exposure to air during movement. The change is large (33.5% when going from 1 to 5 cm), so automated systems clearly report it. The increase in static charges with increasing pressure is due to the increased compressing of the textile structure, which causes more inter-fiber friction. Over one bar, the change in static charges is not so large anymore, so over a threshold pressure, the influence of pressure will not be large anymore. 

The rise in electrostatic charges with larger sample sizes is due to increased material exposure over a larger surface area, with the influence being largest when going to very small samples, so testing should always be done on a sufficiently large testing sample to be representative. The same applies in case of testing time; it should be sufficiently large to be able to reach the saturation point as 60 s in this research testing. The validation performed in this research gives clear guidelines on settings to use and what parameters are important to report on when using automated static testing devices. The devices must be used over a testing time of a minimum of 1 min, must step on samples being 1 m^2^ or higher, must use pressure higher than 1 bar, and dwell time and stepping height must be clearly reported as these factors have an effect on the generation of static charges. Table 7 summarized the effect of different parameters on the overall electrostatic charge generation. 

## Figures and Tables

**Table 1:** Effect of different frequency on the electrostatic charge generation.

Frequency	Repeats	*n* (Number of Peaks)	Peak Voltage (V)	CV %	Peak to Peak Voltage (V)	CV %
1 Hz	3	15	742	5.03	506	3.76
1.5 Hz	3	15	822	2.53	551	6.16
2 Hz	3	15	1186	3.80	846	4.03

**Table 2:** Effect of different pressure on the electrostatic charge generation.

Pressure	Repeats	*n* (Number of Peaks)	Peak Voltage (V)	CV %	Peak to Peak Voltage (V)	CV %
0.5 Bar	3	15	334	13.35	154	3.37
1 Bar	3	15	477	6.25	242	5.78
1.5 Bar	3	15	524	2.89	268	1.15

**Table 3:** The effect of the testing duration on the electrostatic charge generation.

Time	Repeats	*n* (Number of Peaks)	Peak Voltage (V)	CV %	Peak to Peak Voltage (V)	CV %
10 s	3	15	376	5.32	340	3.60
20 s	3	15	717	4.05	548	5.59
30 s	3	15	948	2.85	708	2.62
40 s	3	15	1141	1.48	840	1.28
50 s	3	15	1240	2.49	905	1.61
60 s	3	15	1313	0.38	919	0.11

**Table 4:** Effect of stepping height on the electrostatic charge generation.

Stepping Heights	Repeats	*n* (Number of Peaks)	Peak Voltage (V)	CV %	Peak to Peak Voltage (V)	CV %
10 mm	3	15	651	4.00	450	4.45
30 mm	3	15	740	2.28	478	2.90
50 mm	3	15	979	3.10	630	3.76

**Table 5:** The effect of the dwell time on the electrostatic charge generation.

Dwell Time (ms)	Repeats	*n* (Number of Peaks)	Peak Voltage (V)	CV %	Peak-to-Peak Voltage (V)	CV %
100	3	15	883	0.22	551	1.51
200	3	15	724	2.66	538	1.98
300	3	15	574	4.31	481	4.79

**Table 6:** Effect of sample size on the electrostatic charge generation.

Sample Size	Repeats	*n* (Number of Peaks)	Peak Voltage (V)	CV %	Peak-to-Peak Voltage (V)	CV %
64 cm^2^	3	15	312	0.75	92	7.21
0.5 m^2^	3	15	1328	0.70	96	13.29
1 m^2^	3	15	1538	4.10	131	11.01

**Table 7:** Effect of different parameters on the electrostatic charge generation.

Sr. No.	Different Parameters	Electrostatic Charges Generation
1	Frequency	Increase with increasing frequency
2	Pressure	Increase with the increase in pressure for the compressible sample
3	Time duration	Increase with the increase in time until a saturation point.
4	Stepping height	Increase with the increase in stepping height
5	Dwell time between moves	Decrease with the increase in dwell time at same frequency
6	Sample size	Increase with the increase in sample size

## Data Availability

Most of data is presented in this article. More detailed data presented in this study are available on request from the corresponding author.
